# Effects of Cyclophosphamide Administration on Wool Quality, Physiological and Biochemical Parameters, Carcass Traits, and Meat Quality in Sheep

**DOI:** 10.3390/ani16050756

**Published:** 2026-03-01

**Authors:** Yifan Hu, Peiling Wei, Ping Gong, Xuefeng Lv, Yaqin Wu, Wenping Dong, Rongyin Zhang, Xin Hu, Wenxin Zheng

**Affiliations:** 1 College of Animal Science, Xinjiang Agricultural University, Urumqi 830052, China; 15083313151@163.com; 2 Xinjiang Uyghur Autonomous Region Academy of Animal Science, Urumqi 830011, China; 15699193535@163.com (P.W.); ggpp99@foxmail.com (P.G.); lxf00700@163.com (X.L.); huaer901@163.com (R.Z.); huxin333@163.com (X.H.); 3 Institute of Animal Husbandry Quality Standards, Xinjiang Academy of Animal Sciences, Urumqi 830000, China; 18189636538@163.com (Y.W.); 18394493921@163.com (W.D.)

**Keywords:** cyclophosphamide, Chinese merino sheep, carcass traits, amino acids, fatty acids, wool quality

## Abstract

Chemical depilation presents a promising alternative to traditional shearing for Chinese Merino sheep, aiming to overcome issues of low efficiency and animal stress. Nonetheless, the safety of meat derived from sheep treated with this technology requires comprehensive scientific evaluation. Therefore, this study aimed to investigate the effects of CPA on wool quality, physiological and biochemical parameters, carcass traits, meat quality, fatty acids, and amino acids in sheep. Sheep were treated with different concentrations of CPA for 30 days, and slaughtering was performed at 3, 15, and 30 days post-administration. The CPA treatment (25–30 mg/kg) achieved complete defleecing and significantly increased staple and stretch lengths without affecting fiber length. At the 25 mg/kg dose, certain physiological and biochemical parameters, including mean corpuscular volume, mid-cell count, and lymphocyte percentage, recovered over time, with only short-term fluctuations observed. Moreover, treatment with 25 mg/kg CPA did not affect carcass characteristics, muscle amino acid composition, or meat quality parameters. In conclusion, this study verifies that complete wool shedding can be achieved with CPA at 25–30 mg/kg, while meat quality remains unaffected, providing a scientific basis for its adoption in dual-purpose sheep production.

## Simple Summary

Traditional shearing causes mechanical skin damage and induces stress responses, indicating the need for a better alternative shearing method. In this study, we investigated the effects of cyclophosphamide (CPA), a chemical shearing agent, on the wool quality, performance, and meat quality of Chinese Merino sheep. Our study suggested that CPA treatment at doses of 25–30 mg/kg effectively induced wool defleecing and increased wool length in Chinese Merino sheep. At the 25 mg/kg dose, some physiological and biochemical parameters showed short-term fluctuations but remained within the normal range and recovered over time. Carcass traits, meat quality, and muscle amino acid composition showed no significant changes following CPA treatment, and key fatty acids associated with mutton odor remained stable. Our study provides evidence for the application of CPA in dual-purpose sheep production (meat and wool).

## 1. Introduction

Sheep wool is a natural and renewable textile material that plays an important role in the textile industry and is widely used in clothing, blankets, and household textiles [1]. Current wool harvesting primarily relies on manual [2], electric [3], and mechanical shearing [4]. However, these methods have some limitations. Manual and electric shearing are labor-intensive and have limited efficiency [5]. Mechanical shearing involves expensive equipment and is constrained by pastoral operating conditions [4], making large-scale implementation difficult. Therefore, traditional shearing methods cannot simultaneously meet the comprehensive needs of modern animal husbandry in terms of efficiency, cost, and animal welfare [6]. In recent years, biological defleecing technology has gradually emerged as a potential alternative to traditional shearing, including the use of chemical and biological agents such as cyclophosphamide (CPA) and epidermal growth factor [7], to induce hair follicles into the telogen phase for natural defleecing [8]. CPA offers the advantages of controllable costs and convenient acquisition, making it suitable for pastoral production conditions [9]. Although these technologies have shown good defleecing effects in sheep, goats, and rabbits [10], systematic research on their effects on meat quality, food safety, and potential physiological impacts remains limited. Moreover, their mechanisms of action are not fully understood.

Cyclophosphamide has consistently been a subject of research interest. Cyclophosphamide (CPA) has a limited availability of historical data for use in production animals. However, extensive research across multiple non-production experimental species—such as rats, mice, rabbits, dogs, and even amphibians—has systematically delineated its toxicological profile, safety thresholds, and spectrum of biological effects [11,12]. For example, 15 mg/kg/day is established as a safe dose in rats [13], while single or short-term doses of 50–100 mg/kg are used to induce an immunosuppressed state in mice [14]. In the instructions for cyclophosphamide, there is a 39-week, long-term toxicity study in dogs. This study clearly defines a dose of ≤20 mg/kg/day as the safe range. A dose-escalation study aimed at determining the maximum tolerated dose (MTD) further confirmed that, when supported by autologous bone marrow transplantation, the intravenous MTD of cyclophosphamide can reach 500 mg/m^2^, with manageable toxicity [15]. This study concludes that 20–30 mg/kg is within a reasonable dosage range, and it is not administered daily but as a single dose. Building upon this broad background, the present study aims to obtain more comprehensive and reliable safety data. Recent investigations have primarily focused on its mechanisms of ovarian protection [16], intestinal barrier function repair [17], and immunomodulatory effects [18]. Although its physiological and biochemical impacts have been relatively well-elucidated [19], the effects of cyclophosphamide on animal slaughter performance and meat quality remain underexplored. Existing studies indicate that cyclophosphamide does not significantly affect the growth performance of broilers [20]; however, systematic studies regarding its influence on meat quality characteristics, amino acid composition, and fatty acid profiles in ruminants (such as sheep) are still scarce.

Therefore, it is necessary to determine the effects of CPA on meat quality and nutritional composition before applying it as a defleecing agent in sheep. CPA functions as an alkylating agent precursor [21], and its metabolites can induce apoptosis by disrupting DNA structure [22,23]. Research on post-CPA defleecing physiological responses, slaughter performance, and meat quality changes in sheep is limited. For biological defleecing technology to become a viable alternative to traditional shearing, safety and product quality must be ensured. This study employed targeted mass spectrometry technology to systematically analyze changes in the meat quality (amino acid and fatty acid composition) of Chinese Merino sheep following cyclophosphamide (CPA) injection, and comprehensively evaluated wool quality, physiological and biochemical indicators, as well as slaughter performance. The aim was to provide a holistic assessment of the safety and applicability of CPA as a depilatory agent in sheep, thereby offering a scientific basis for its practical production application.

## 2. Materials and Methods

### 2.1. Ethics

We used 56 Chinese Merino sheep in this study. When collecting blood, we maintained aseptic procedures to reduce discomfort to the sheep. Humane measures, such as behavioral calming, were used to reduce animal stress and discomfort before slaughter. Animals were stunned electrically or mechanically before slaughter to avoid suffering. All procedures followed strict aseptic principles. All animal experiments strictly adhered to animal welfare ethics standards, maximizing respect and protection of animal welfare, ensuring all animals were free from unnecessary pain and suffering during the experiment. All experimental procedures were reviewed and approved by the Animal Ethics Committee of the Institute of Animal Husbandry Quality Standards, Xinjiang Academy of Animal Sciences (No.: 20250512034).

### 2.2. Experimental Design

A total of 56 half-sibling Chinese Merino ewes aged 12 months with similar body weights, none of which had been shorn, were used in this study. This study was conducted in two batches using a completely randomized design. In the first batch, sheep were treated with different concentrations of CPA (*n* = 8 sheep/group): 0 (control), 20, 25, and 30 mg/kg. In the second batch, sheep were assigned to control (0 mg/kg CPA; 6 sheep) and experimental groups (25 mg/kg CPA; 18 sheep) for 30 days.

To evaluate blood physiological indices and wool quality, the first cohort of 32 sheep was randomly divided into four groups (*n* = 8/group), including a control group (*n* = 8/group). On day 30 of the trial, shearing was performed by skilled personnel familiar with the animals. Whole blood samples (3 mL) were collected aseptically from the jugular vein at 0, 5, 7, 15, and 30 days post-injection. For the assessment of blood biochemical parameters, slaughter performance, meat quality, and amino and fatty acid composition, a second cohort of 24 sheep was divided into four groups (*n* = 6/group), including both experimental and control groups (*n* = 6/group). During the 30-day period, six sheep were randomly slaughtered at 3, 15, and 30 days post-injection in the experimental group. Animals in the control group were uniformly slaughtered at 30 days of the experiment. All measurements were compared with the aforementioned shared control group. The sample size was determined with reference to similar goat hair removal experiments and in compliance with animal ethics guidelines [24].

### 2.3. Feeding and Management

All sheep were raised at a farm in Baicheng County, Xinjiang, under unified management during the feeding period. Feeding, feeding schedules, and environmental conditions remained unchanged before and throughout the experiment. During the pre-trial period, sheep pens were thoroughly cleaned and disinfected, ewe ear tags were verified, and grouping was performed. Following the grouping process, the ewes were placed into pens according to their respective groups. Transitional feeding was conducted during the pre-trial period to reduce stress responses in sheep. The formal experiment began on 25 April 2025, with feeding at 08:00 and 17:00 daily, allowing ad libitum feeding and drinking. The basal diet was a pelleted feed formulated according to “Nutrient Requirements of Meat-producing Sheep and Goats” (NY/T 816-2021) [25], with a forage-to-concentrate ratio of 60:40. The composition of the basic diet is shown in Table 1. 

The premix provided the following per kg of the diet: VA 15 000 IU, VD 2 500 IU, VE 40 IU, Cu 15 mg, Zn 25 mg, Fe 55 mg, Se 0.3 mg, Mn 40 mg, Co 0.2 mg, and I 0.5 mg.

### 2.4. Sample Collection

On day 30 post-injection, 20 cm^2^ areas were marked on both sides of the body (approximately one palm width behind the scapula, slightly above the body midline) and on the thigh (midpoint of the line connecting the tuber coxae and hock) of sheep in the first batch, and approximately 150 g of wool samples were collected close to the skin for determination of fiber length, diameter, color, and clean wool rate [26]. Additionally, 3 mL of whole blood was collected from the jugular vein before morning feeding at 0, 5, 7, 15, and 30 days post-injection. One portion was used for the immediate analysis of physical and chemical parameters, whereas the other portion was centrifuged to separate serum, which was stored at −80 °C for future use.

For the second batch, six sheep in the 25 mg/kg CPA group were humanely slaughtered at 3, 15, and 30 days post-administration. Sheep were fasted for 12–24 h and deprived of water for 3 h before slaughter, and the fasting live weight was recorded. After slaughter, carcass weight was measured. Thereafter, 15 × 5 cm *longissimus dorsi* muscle samples were collected between the 12th and 13th ribs. A portion of the muscle sample was used for the analysis of physical and chemical parameters, whereas the other portion was stored at −80 °C for future use. Slaughter was performed by licensed veterinarians using electrical stunning, followed by exsanguination, complying with animal ethics standards.

### 2.5. Main Reagents and Equipment

Main experimental materials and consumables included CPA solution (Baxter Oncology GmbH, Halle, Germany, 3I633A), veterinary blood cell analysis hemolytic agent (Jiangxi Tekang Technology Co., Ltd., Nanchang, China, VB2203002), and veterinary blood cell analysis diluent (Jiangxi Tekang Technology Co., Ltd., V-3D).

Equipment used in this study included OFDA2000 fiber fineness analyzer (IWG, Sydney, Australia, OFDA2000), semi-automatic four-tank wool washing equipment (J.GOODWIN, W/4-17), blood cell analyzer (Jiangxi Tekang Technology Co., Ltd., TEK-VET3), liquid chromatograph (AB SCIEX, ExionLC model), mass spectrometer (AB SCIEX, Framingham, MA, USA, AB 6500 Plus), gas chromatograph system (Thermo, Waltham, MA, USA, TRACE1300), and mass spectrometer (Thermo, TSQ9000).

### 2.6. Measurement Indicators and Methods

#### 2.6.1. Blood Physiological Parameters

Whole blood parameters, including white blood cell count (WBC, 10^9^/L), red blood cell count (RBC, 10^12^/L), hemoglobin (HGB, g/L), mean corpuscular volume (MCV, fL), lymphocyte count (Lym, 10^9^/L), mid-cell count (Mid, 10^9^/L), lymphocyte percentage (Lym, %), and mid-cell percentage (Mid, %), were analyzed measured using an automatic blood cell analyzer (PE-6800VET: Shenzhen Pukang Electronic Co., Ltd., Shenzhen, China) [27].

#### 2.6.2. Blood Biochemical Parameters

Serum parameters, including creatine kinase content, creatine kinase activity, and myoglobin, were analyzed using specific kits. Briefly, the color depth of serum myoglobin (MYO/MB) and creatine kinase (CK) levels was measured using assay kits based on the double antibody sandwich enzyme-linked immunosorbent assay (ELISA) method. Finally, the absorbance (OD value) was measured at 450 nm using a microplate reader, and MYO/MB ratio, CK levels, and CK activity were calculated using standard curves. All kits were purchased from Norminkoda Biotechnology Co., Ltd. (Wuhan, China).

#### 2.6.3. Wool Quality

After routine washing, samples were naturally dried. All parameters were measured in a constant temperature and humidity environment (20 ± 2 °C; relative humidity, 65 ± 4%). Fiber diameter was determined using the Fiber Diameter Optical Analyzer Method according to GB/T21030-2007 “Test Method for Mean Diameter and Distribution of Wool and Other Animal Fibers” [28]. Clean wool rate was determined using the GB1523-2013 “Sheep Wool” clean wool rate measurement method [29]. Hand-stapled length and single fiber length were measured using the GB18267-2013 “Cashmere” hand-stapled length test method [29]. Color was determined using the IWTO-56-03 “Method for Determination of Color of Raw Wool” [30].

#### 2.6.4. Carcass Traits

Pre-slaughter live weight was measured before slaughter, after 24 h of fasting, and 3 h of water deprivation. Carcass weight was measured post-slaughter after removing the head, hooves, hide, blood, and internal organs (except kidneys and surrounding fat).

Dressing percentage (DP, %) was calculated as

DP = (W_carcass/W_live) × 100%

where W_carcass is the hot carcass weight (kg), and W_live is the live weight at slaughter (kg).

#### 2.6.5. Meat Quality

Crude fat content was determined using Soxhlet extraction according to GB5009.6-2016 “National Food Safety Standard, Determination of Fat in Foods” [31]. Crude protein content was analyzed using the Kjeldahl method according to GB5009.5-2016 “National Food Safety Standard, Determination of Protein in Foods” [32]. pH measurement was performed three times per sample using a pH meter according to the NY/T1121.2-2006 standard procedure, and the average was calculated [33]. Shear force was measured using a RapidTA texture analyzer (manufactured by Shanghai Tengbo Instrument Technology Co., Ltd., Shanghai, China). Marbling score was graded on a 5-point scale under indoor natural light conditions by comparing meat cross-sections to American pork marbling standard charts.

Meat color evaluation was conducted under indoor natural light using visual assessment with a 5-grade standard chart. Lightness (L*), redness (a*), and yellowness (b*) were measured at 45 min and 24 h post-slaughter using a portable colorimeter according to NY/T2793—2015 “Objective Evaluation Method for Eating Quality of Meat” [34]. Each sample was measured three times at different locations, and the results were averaged. Water holding capacity (WHC, %) was calculated as

WHC = [1 − (W_1_ − W_2_)/W_1_] × 100%

where W_1_ is the pre-centrifugation meat weight (g), and W_2_ is the post-centrifugation meat weight (g).

Moisture content was determined by drying at 101–105 °C and 101.3 kPa until a constant weight was attained according to GB 5009.3-2016 “National Food Safety Standard—Determination of Moisture in Foods” [35].

#### 2.6.6. Amino Acids

Amino acids and fatty acids were detected using specific methods. For amino acid analysis, standard substances were precisely weighed to prepare single standard stock solutions and Trp-d_3_ internal standards, which were mixed to obtain working solutions. Samples were ground, extracted with 10% formic acid in methanol–water, and centrifuged to obtain the supernatant, which was diluted 50-fold. An internal standard was added, and samples were filtered for injection [36,37]. An ACQUITY UPLC^®^ BEH C18, chromatographic column (2.1 × 100 mm, 1.7 μm, Waters, Milford, MA, USA) was used with gradient elution using methanol–water containing formic acid. Mass spectrometry conditions used an electrospray ionization (ESI) source in positive ion mode, followed by multiple reaction monitoring (MRM) scanning [38,39]. Finally, amino acid content was calculated using the following formula: amino acid (μg/g) = C (ng/mL) × 0.6 × 2 × 50/sample weight (mg).

#### 2.6.7. Fatty Acids

Gradient working solutions were prepared using fatty acid methyl ester mixed standards. Samples were extracted with chloroform–methanol, ultrasonically treated, centrifuged, esterified in a water bath, extracted with n-hexane, washed with water, dehydrated, diluted, and methyl salicylate internal standard was added [30]. Fatty acid content was determined using a gas chromatography system (TG-FAME column with programmed temperature) coupled with a mass spectrometer (electron impact ionization EI source with selected ion monitoring SIM scanning). Finally, fatty acid content was calculated using the following formula: fatty acid (μg/g) = C (ng/mL) × 1 × 10/sample weight (mg) × 1000 [40].

### 2.7. Data Analysis

All data were organized using Microsoft Excel 2021, and all statistical analyses were performed using SPSS 21.0 software (IBM Corp.). Prior to analysis, the normality of data distribution and homogeneity of variances were assessed using the Shapiro–Wilk test and Levene’s test, respectively. For data that satisfied both assumptions, one-way analysis of variance (ANOVA) was employed for inter-group comparisons, with the treatment group (cyclophosphamide dosage or duration) as a fixed factor. When a significant main effect was detected, Tukey’s honest significant difference (HSD) post hoc test was applied for multiple comparisons. These data are presented as mean ± standard error of the mean (SEM). For data that violated either assumption, non-parametric tests were used. The Kruskal–Wallis H test was applied for overall inter-group comparisons. If a significant difference was detected, Dunn’s post hoc test was used for pairwise comparisons. These data are presented as median (interquartile range). The level of statistical significance was set at *p* < 0.05. A value of 0.05 ≤ *p* < 0.1 was considered to indicate a statistical trend, whereas *p* ≥ 0.1 was considered not significant.

## 3. Results

### 3.1. Effects of CPA on Defleecing Efficacy and Wool Quality in Sheep

**Figure 1:**
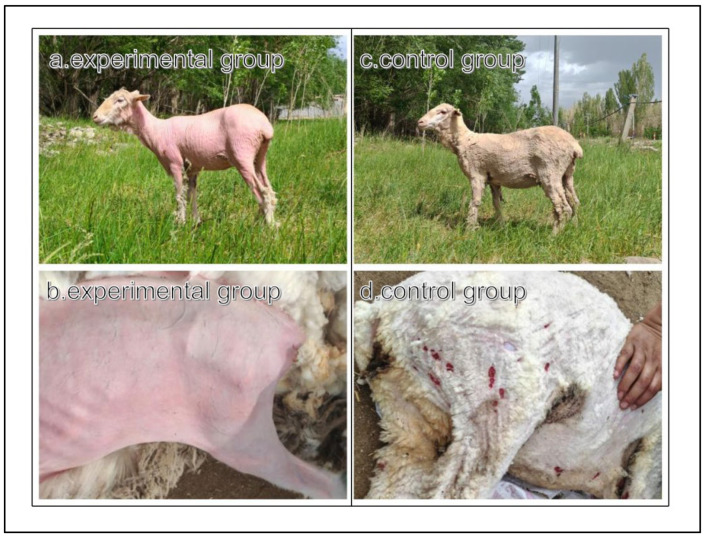
Comparison of biological defleecing and electric shearing.

Chinese Merino sheep treated with CPA were defleeced within the specified time (Figure 1). Notably, CPA-induced defleecing was thorough, with intact skin surfaces showing no cuts or need for secondary trimming (Figure 1a,b). During the CPA treatment period, sheep behavior remained stable, with no obvious stress responses, thus meeting animal welfare requirements. In contrast, sheep in the electric shearing group exhibited varying degrees of skin cuts accompanied by mild stress behavior (Figure 1c,d). Additionally, staple and stretched lengths were significantly higher (*p* < 0.05) in the CPA group than in the shearing group (Table 2). However, no significant differences were observed in wool diameter, clean wool rate, or color parameters between the two groups.

This discovery holds significant practical value as an increase in hair length directly indicates an increase in the single production yield of wool, potentially leading to economic benefits. It is worth noting that although the hair length has significantly increased, there are no significant differences between the two groups in key indicators that determine the commercial value of wool, such as wool fineness, net wool yield, and color. This suggests that cyclophosphamide depilation not only enhances the yield but also does not sacrifice the basic quality of the wool.

### 3.2. Effects of CPA on Physiological and Biochemical Parameters in Sheep

#### 3.2.1. Effects of CPA on Blood Physiological Parameters

As shown in Table 3, Chinese Merino sheep in the 25 mg/kg cyclophosphamide group exhibited changes in the mean red blood cell volume (MCV) and intermediate cell count (Mid) over time. Although these parameters fluctuated, none of the differences reached statistical significance compared to the control group, and all values remained within the normal physiological range. More importantly, key immune function indicators, including white blood cell count (WBC), lymphocyte count (Lym), and their percentage (Lym%), showed no significant differences across all treatment groups and time points (*p* > 0.05). This indicates that cyclophosphamide, at the 25 mg/kg dose, did not exert biologically significant toxicity on the sheep’s hematopoietic system or basic immune function, demonstrating its favorable safety profile.

#### 3.2.2. Effects of CPA on Blood Biochemical Parameters

The 25 mg/kg CPA treatment triggered a reversible increase in key muscle metabolism markers (CK and myoglobin) by day 15, which normalized by day 30 (Table 4). This transient response indicates that cyclophosphamide’s effect on muscle is temporary and self-limiting, a crucial point for its safety assessment.

### 3.3. Effects of CPA on Carcass Traits in Sheep

The slaughter performance of sheep remained stable following CPA treatment. As detailed in Table 5, pre-slaughter live weight, carcass weight, and dressing percentage showed no significant changes at 3, 15, and 30 days post-administration compared to the control group (*p* > 0.05). Most importantly, this stability in key production metrics indicates that the depilation process induced by CPA does not compromise the core economic value of the animals.

### 3.4. Effects of CPA on Meat Quality in Sheep

The impact of 25 mg/kg CPA on *longissimus dorsi* muscle quality was found to be limited and transient. Although treatment with 25 mg/kg CPA caused significant inter-group differences in the pH and yellowness (b*) of the *longissimus dorsi* muscle at 3, 15, and 30 days post-administration (*p* < 0.05), the parameters remained within the normal physiological range (Table 6). This indicates that the observed changes, while detectable, did not reach a level indicative of meat quality deterioration. Specifically, the pH and yellowness values showed a quadratic pattern in the 25 mg/kg CPA group, peaking at 30 and 15 days post-treatment, respectively, and then decreasing. Moisture, crude protein, and crude fat contents, shear force, marbling score, water holding capacity, meat color grade, and lightness (L*) and redness (a*) values showed no significant differences among the groups (*p* > 0.05).

### 3.5. Effects of CPA on Amino Acids in Sheep

**Figure 2:**
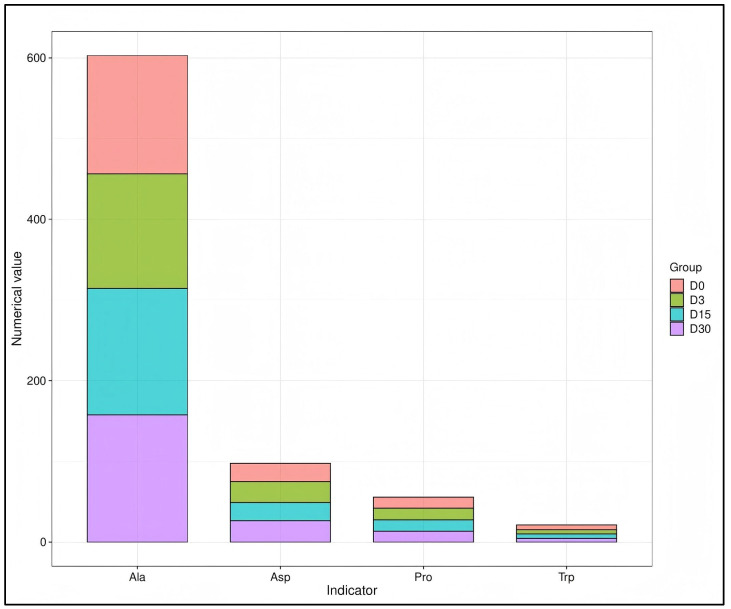
Effects of cyclophosphamide (CPA) on the levels of flavor amino acids in sheep. D0 represents the control group. D3, D15, and D30 represent the 25 mg/kg CPA group slaughtered at 3, 15, and 30 days post-treatment. Ala: alanine; Asp: aspartic acid; Pro: proline; Trp: tryptophan.

The amino acid composition of the *longissimus dorsi* muscle in Chinese Merino sheep remained stable following CPA treatment. This compositional stability is a key determinant of the meat’s nutritional value and flavor potential. Treatment with 25 mg/kg CPA did not significantly affect (*p* > 0.05) essential and non-essential amino acid contents in the *longissimus dorsi* muscle of Chinese Merino sheep at 3, 15, and 30 days post-administration (Figure 2; Table 7). Similarly, no significant differences were observed in the levels of flavor-related amino acids, including tryptophan, alanine, proline, and aspartic acid, among the groups (*p* > 0.05).

### 3.6. Effects of CPA on Fatty Acids in Sheep

**Figure 3:**
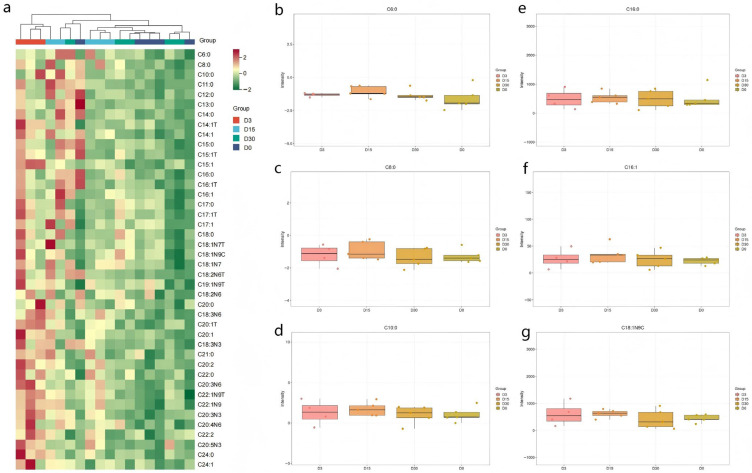
(**a**–**g**) Effects of cyclophosphamide on fatty acid content in Chinese Merino sheep. D0 represents the control group. D3, D15, and D30 represent the 25 mg/kg experimental group slaughtered at 3, 15, and 30 days post-treatment, respectively.

The impact of 25 mg/kg CPA on the fatty acid profile of the *longissimus dorsi* muscle in Chinese Merino sheep was limited and transient, with no adverse effects on key quality attributes. CPA treatment had no significant effect (*p* > 0.05) on the levels of saturated fatty acids in the *longissimus dorsi* muscle of Chinese Merino sheep (Table 8). As shown in Figure 3a, changes in fatty acid levels mainly manifested as short-term fluctuations that gradually recovered after day 15 post-administration, approaching control levels by day 30. Moreover, no significant differences (*p* > 0.05) were observed in the levels of major fatty acids associated with mutton odor (C6:0, C8:0, and C10:0) and meat quality (C16:0, C16:1, and C18:1N9C) among the groups (Figure 3b–g). In contrast, treatment with 25 mg/kg CPA significantly increased the levels of some unsaturated fatty acids, including C15:1, C20:1T, C22:1N9, C22:1N9T, C20:2, and C20:3N3, in Chinese Merino sheep at 3 days post-treatment compared with those in the control and other CPA groups (Table 9 and Table 10).

## 4. Discussion

Heat stress has become an important environmental factor constraining livestock production, particularly under intensive farming conditions [41]. Research has shown that sheep shearing can affect thermoregulatory responses in sheep [42], reducing thermal insulation, increasing heat loss, and decreasing rectal temperature [43]. Additionally, shearing can reduce surface temperature [44], benefiting thermoregulation in tropical environments [45]. As animals lacking the natural ability to shed wool, sheep have long relied on manual or mechanical shearing for wool harvesting; however, post-shearing sheep experience varying degrees of stress [46]. In recent years, chemical shearing has emerged as a safe alternative due to increasing animal welfare awareness [47]. Traditional shearing has disadvantages, such as skin cuts, sheep stress, and poor wool quality. CPA is an effective defleecing agent that can achieve damage-free defleecing [48]. Studies in mouse models have demonstrated that cyclophosphamide treatment effectively induces hair loss [49], with no adverse effects observed on the tensile strength of intact skin, the strength of damaged skin, or the torsional strength of the femoral shaft [50].

This study systematically evaluated the efficacy and safety of cyclophosphamide (CPA, 25–30 mg/kg) as a depilatory agent for sheep. The core results indicated that 25 mg/kg CPA achieved effective depilation while also enhancing wool quality, without causing significant negative impacts on the physiological health, slaughter performance, and nutritional quality of lamb meat. The efficacy might be attributed to CPA’s anti-mitotic effect on rapidly dividing cells at the base of hair follicles, which can reversibly inhibit hair fiber growth and promote their shedding [51]. In light of the absence of negative impacts on key economic traits such as slaughter performance and meat quality, it is reasonable to speculate that CPA at this dosage exhibits high tissue specificity, with its pharmacological effects confined locally to the hair follicles and without triggering systemic toxicity.

Wool, a structurally complex natural protein fiber, possesses excellent insulation, flame resistance, durability, and hygroscopicity, and is widely used in clothing and other manufacturing industries [52]. Fiber diameter, length, yield, and clean wool rate are key phenotypic traits of wool fibers that directly affect their economic value [53]. Research has shown that CPA defleecing can significantly increase wool yield compared with traditional shearing [24], which was confirmed in the present study. Treatment with 25 and 30 mg/kg CPA achieved complete defleecing, whereas the 20 mg/kg dose showed partial defleecing. Similarly, a previous study showed that 27.0 mg/kg CPA can achieve stable wool shedding in sheep [54]. In the present study, CPA-treated sheep showed no skin damage, need for secondary trimming, or any obvious behavioral abnormalities. In contrast, electrically shorn sheep showed varying degrees of skin cuts and mild stress. Given that CPA treatment increased wool length without affecting diameter [55], clean wool rate, or color, it can be concluded that CPA defleecing improves the commercial characteristics of wool and meets current animal welfare requirements in livestock production.

Given the known cytotoxicity of CPA and its potential bone marrow suppressive effects, its impact on physiological homeostasis should be considered for application in livestock production. Previous studies have indicated that CPA affects routine blood parameters [56]. For instance, CPA significantly reduced RBC and HGB levels in sheep at doses of 20–25 mg/kg [24]. In the present study, most blood physiological parameters remained within the normal range throughout the observation period, with no significant differences between the treatment and control groups. This may be associated with low-dose administration. In high-dose myelosuppressive regimens (typically ≥100 mg/kg), the active metabolites of cyclophosphamide induce severe DNA cross-linking, leading to apoptosis of rapidly proliferating hematopoietic progenitor cells and consequently sustained pancytopenia [57]. In contrast, the lower-dose, immunomodulatory regimen employed in this study (25 mg/kg) likely operates below a distinct threshold. Furthermore, sustained low-dose exposure may even trigger a compensatory stress response in the bone marrow, promoting rapid recovery [58], which also explains the reversible nature of the observed changes. Based on the above results, it can be speculated that at the experimental dose and conditions of this study, the impact of low-dose cyclophosphamide on the hematopoietic system is relatively mild.

Creatine kinase and myoglobin are important markers of skeletal muscle damage. Although CK content, myoglobin level, and CK activity exhibited temporary increases in the 25 mg/kg CPA group at 15 days post-administration, these parameters recovered to near control levels by day 30. This aligns with research showing that rhabdomyolysis caused by high-dose cyclophosphamide is reversible within two weeks upon drug withdrawal [59]. Previous studies have indicated that high doses or long-term use of CPA may increase CK activity and myoglobin levels [60], potentially leading to rhabdomyolysis in severe cases [61]. Importantly, the changes observed in this study were consistent with the characteristics of short-term muscle stress responses, without accompanying abnormal changes in slaughter performance or meat quality.

From a production perspective, carcass traits are important indicators of the economic value of sheep [62,63]. Our study showed that treatment with 25 mg/kg CPA had no significant effects on pre-slaughter live weight, carcass weight, and dressing percentage at 3, 15, and 30 days post-administration, indicating that CPA had no adverse effects on short-term production performance. This result is important because drug metabolism or tissue repair processes theoretically cause energy and nutrient redistribution; however, CPA had no relevant negative effects on carcass characteristics.

Meat quality is a key determinant of consumer acceptance and market value of meat products. Consumers typically focus on quality characteristics such as meat color, marbling, tenderness, and flavor [64]. Among these, meat color is the most intuitive indicator for evaluating meat quality, marbling directly affects sensory scores [65], and pH is closely related to muscle water-holding capacity and color [66], which are all important factors in determining meat quality [67]. In the present study, CPA treatment did not significantly affect the moisture, crude protein, and crude fat contents, shear force, marbling score, water holding capacity, and objective color parameters (L* and a*) of the *longissimus dorsi* muscle. Although pH and b* showed statistical differences at some timepoints, these changes were limited and remained within the normal range.

Cyclophosphamide (CPA) is a prodrug whose pharmacological and toxicological effects depend on hepatic activation via the cytochrome P450 enzyme system (particularly CYP2B6), generating active metabolites such as 4-hydroxycyclophosphamide. After these active metabolites are distributed throughout the body via the bloodstream, their main cytotoxic mechanism is to cause DNA alkylation, forming inter-strand cross-linking damage to DNA, and ultimately activating the caspase 9-dependent apoptotic pathway [68], thereby exerting a significant effect on rapidly dividing cells [69]. In this study, CPA did not cause significant adverse effects on sheep skeletal muscles (e.g., the *longissimus dorsi*). This result can be reasonably explained by the tissue specificity of the CPA action mechanism. The skeletal muscles of adult sheep belong to terminally differentiated tissues with extremely low cell division activity [70], so they are relatively insensitive to the active metabolites of CPA that mainly target rapidly proliferating cells. In contrast, hair follicle cells have vigorous cell division, thus becoming the target of CPA’s depilation effect, which further demonstrates the selectivity of its action.

Amino acid composition is an important indicator of meat nutritional value and flavor. Amino acids are critical for maintaining health and production performance [71], and their composition, along with that of fatty acids, jointly affects meat quality characteristics and animal health [72]. Research has shown that alanine, aspartic acid, and glutamic acid contribute to the umami flavor of meat [73]. Among these, glutamic acid and aspartic acid can suppress undesirable flavors, such as saltiness and sourness [74], and alanine can stimulate taste buds to produce sweetness and mitigate the bitterness and saltiness of some amino acids [75]. Arginine participates in protein deposition processes, thereby affecting meat quality [76]. Our study showed that CPA treatment had no significant effects on essential and non-essential amino acid contents in lamb, including various amino acids closely related to umami flavor and taste. Overall, these results indicate that CPA defleecing treatment at the studied dose does not interfere with muscle protein deposition and amino acid composition, thereby ensuring the basic nutritional quality of lamb meat.

From the perspective of a mechanism, this study found that CPA treatment did not significantly alter the amino acid profile of lamb meat. This stability can be speculated to be caused by the tissue specificity of CPA action [77]. A deeper reason might lie in the powerful endogenous antioxidant system of skeletal muscle cells [78]. The reactive intermediates (such as acrolein) produced during cyclophosphamide metabolism can induce oxidative stress [79]. Glutathione (GSH), as the most important endogenous antioxidant and detoxification molecule in cells, shows a significantly activated state in muscle and other highly metabolically active tissues [80]. We speculate that skeletal muscle cells can effectively neutralize the mild oxidative stress induced by CPA by maintaining sufficient GSH levels, thereby protecting cellular structures, including the protein synthesis system, from oxidative damage. The amino acid profile and content of the muscle are sensitive indicators reflecting the status of protein metabolism and deposition, which provides a mechanistic explanation at the cellular level for the stability of the amino acid profile.

The fatty acid composition is directly related to the flavor and lipid nutritional structure of mutton. Research has shown that unsaturated fatty acids, such as linoleic acid, reduce the risk of cardiovascular disease in humans [81]. Additionally, saturated fatty acids, such as C18:0, are positively correlated with mutton off-flavor intensity [82]. C8:0, C10:0, and C12:0 are highly correlated with the characteristic ‘mutton odor,’ with higher contents leading to more pronounced undesirable flavors [83]. In the present study, the levels of medium- and short-chain saturated fatty acids (such as C8:0, C10:0, C12:0, and C18:0), which are closely related to mutton odor formation and sensory characteristics, were not significantly different between the CPA and control groups, suggesting that CPA defleecing had no obvious effect on characteristic flavor fatty acids in lamb. Among the unsaturated fatty acids, some fatty acids showed statistical differences, with potentially unfavorable trans fatty acids for human health (C20:1T, C22:1N9, and C22:1N9T) showing downward trends in the treatment group. However, changes in the levels of other fatty acids were minimal, and their nutritional or sensory significance requires further investigation. At the mechanistic level, unsaturated fatty acids are prone to undergo peroxidation reactions and are primary targets of oxidative stress [84,85]. Therefore, the stability of the fatty acid profile serves as strong indirect evidence that the redox homeostasis within muscle cells has not been severely disrupted. This further supports the aforementioned speculation regarding the amino acid profile: that the oxidative stress triggered by CPA at therapeutic doses may fall within the buffering capacity of the glutathione system in muscle cells, thereby protecting the integrity of lipid membranes and lipid droplets. Although the fluctuations in some unsaturated fatty acids are statistically significant, their magnitude of change is small, which may reflect a dynamic adjustment of the GSH system in response to mild stress rather than irreversible oxidative damage.

We acknowledge the limitations of this study. For example, this study was conducted on a single breed under specific feeding conditions. The sample size for this study was determined based on previous similar research. Nevertheless, this sample size may limit the reliability and generalizability of the findings from subgroup analyses, and it may also be underpowered to detect effects of small magnitude. Therefore, the results cannot be directly extrapolated to other breeds or production systems. In terms of the discussion, the proposed role of the glutathione pathway is a reasonable, literature-based speculation. This study did not directly measure the levels of GSH, oxidized glutathione (GSSG), or the activities of related enzymes (such as GPx, GST) in muscle tissue. Therefore, this mechanistic explanation requires verification through future studies involving direct measurement of oxidative stress biomarkers (e.g., MDA) and indicators related to GSH metabolism. Furthermore, there are relatively few specialized studies on the impact of cyclophosphamide on muscle mass. Due to this limitation, the individual viewpoints discussed in this article have not been thoroughly explored or cited. Regarding meat quality, one limitation of this study is that the assessments were conducted on hot carcass or early-postmortem samples without a standardized, extended aging period. This may particularly influence traits such as tenderness and water-holding capacity, which typically evolve during maturation. Our approach was designed to capture the initial effects of the experimental treatment (e.g., CPA administration).

## 5. Conclusions

Our study suggested that CPA injection at doses of 25–30 mg/kg effectively induced defleecing in Chinese Merino sheep. Injecting CPA for hair removal treatment can improve the quality of wool. At the 25 mg/kg dose, some physiological and biochemical parameters exhibited and then recovered from short-term fluctuations. Carcass traits, meat quality, and muscle amino acid composition showed no significant changes following CPA injection, and major fatty acids associated with mutton odor remained stable. In conclusion, CPA defleecing treatment had no significant effects on sheep carcass characteristics or meat quality in this study. However, its long-term safety requires further research.

## Figures and Tables

**Table 1:** Basal diet composition (dry matter basis).

Items	Content (%)
Corn	30.60
Caragana korshinskii	20.00
Soybean meal	12.94
Corn straw	12.00
Oat grass	8.00
Corn germ meal	7.50
Premix	5.00
Sprayed corn bran	1.50
Sunflower seed meal	1.50
Distiller’s grain	0.96
Total	100.00

**Table 2:** Effects of CPA on wool quality in sheep (using the 0 mg/kg control group as the reference).

Dose	Site	Sampling Method	Staple Length (mm)	Stretched Length (mm)	Fiber Diameter (μm)	Clean Wool Rate (%)	Color			Number of Sheep (Group)
							Lightness (L)	Whiteness (Wht)	Yellowness (YI)	
0 mg/kg	Lateral body	Electric shearing	109.33 ± 9.85 BC	139.67 ± 11.45 C	18.37 ± 1.52 a	59.27 ± 7.71 a	72.99 ± 2.3 a	71.93 ± 2.15 a	19.39 ± 1.20 a	8
	Thigh		108.33 ± 18.12 C	140.33 ± 20.36 BC	18.80 ± 1.46 a	60.55 ± 6.89 a	70.70 ± 5.76 a	69.64 ± 5.53 a	19.96 ± 1.44 a	8
20 mg/kg	Lateral body	Cyclophosphamide defleecing	115.50 ± 4.76 BC	143.67 ± 3.78 BC	18.55 ± 0.94 a	61.15 ± 5.35 a	72.21 ± 2.18 a	71.00 ± 2.07 a	20.92 ± 1.66 a	8
	Thigh		115.25 ± 7.25 BC	140.88 ± 11.10 BC	18.75 ± 1.49 a	60.11 ± 3.03 a	70.90 ± 2.32 a	69.83 ± 2.24 a	20.53 ± 1.86 a	8
25 mg/kg	Lateral body	Cyclophosphamide defleecing	130.71 ± 8.56 A	162.14 ± 14.44 A	18.34 ± 1.58 a	62.94 ± 7.18 a	72.38 ± 2.20 a	71.18 ± 2.11 a	20.94 ± 1.45 a	8
	Thigh		123.00 ± 8.51 AB	156.00 ± 12.39 ABC	18.80 ± 1.74 a	62.17 ± 6.72 a	70.54 ± 1.11 a	69.50 ± 1.06 a	20.60 ± 1.53 a	8
30 mg/kg	Lateral body	Cyclophosphamide defleecing	133.20 ± 9.52 A	169.40 ± 6.35 A	18.81 ± 2.83 a	63.27 ± 7.55 a	72.16 ± 2.54 a	71.18 ± 2.61 a	19.68 ± 3.11 a	8
	Thigh		121.60 ± 16.44 ABC	156.70 ± 17.43 AB	18.56 ± 2.71 a	61.34 ± 7.19 a	71.48 ± 3.90 a	68.01 ± 4.88 a	21.15 ± 3.26 a	8

Different uppercase letters in the same column indicate significant differences (*p* < 0.05); same lowercase letters in the same column indicate no significant difference (*p* > 0.05). In this study, the 0 mg/kg group served as the control, providing the baseline normal physiological reference values for this breed under standardized rearing conditions.

**Table 3:** Effects of cyclophosphamide on physiological parameters in sheep.

Indicator	Dose	0D	5D	7D	15D	30D	Number of Sheep (Group)
White Blood Cell Count (WBC)	0 mg/kg	17.20 ± 5.65 a	16.00 ± 2.97 a	17.70 ± 9.25 a	16.30 ± 6.47 a	14.72 ± 4.92 a	8
	20 mg/kg	16.95 ± 6.61 a	16.11 ± 9.41 a	11.18 ± 7.51 a	15.92 ± 4.13 a	15.93 ± 8.14 a	8
	25 mg/kg	16.08 ± 7.74 a	16.36 ± 5.34 a	10.20 ± 3.97 a	15.37 ± 6.17 a	15.14 ± 4.34 a	8
	30 mg/kg	17.22 ± 9.74 a	16.33 ± 7.56 a	10.44 ± 6.28 a	14.85 ± 8.61 a	13.95 ± 6.09 a	8
Red Blood Cell Count (RBC)	0 mg/kg	10.26 ± 1.07 a	10.13 ± 2.44 a	8.67 ± 1.52 a	10.26 ± 0.97 a	10.32 ± 1.31 a	8
	20 mg/kg	9.63 ± 1.40 abc	10.41 ± 0.85 a	8.43 ± 0.89 c	9.08 ± 1.89 bc	10.34 ± 0.95 ab	8
	25 mg/kg	9.59 ± 1.24 a	9.94 ± 0.99 a	8.00 ± 1.56 b	9.44 ± 0.83 a	9.76 ± 1.19 a	8
	30 mg/kg	10.06 ± 0.67 a	10.24 ± 2.22 a	8.74 ± 2.00 a	9.27 ± 2.08 a	9.86 ± 1.02 a	8
Hemoglobin (HGB)	0 mg/kg	116.40 ± 9.61 a	116.67 ± 11.76 a	112.67 ± 9.24 a	117.40 ± 7.92 a	117.80 ± 9.65 a	8
	20 mg/kg	115.82 ± 6.71 a	116.90 ± 6.40 a	107.67 ± 5.28 b	115.20 ± 5.54 a	117.71 ± 5.15 a	8
	25 mg/kg	111.75 ± 7.48 a	116.00 ± 7.39 a	112.60 ± 12.70 a	115.00 ± 7.10 a	118.20 ± 14.29 a	8
	30 mg/kg	114.36 ± 6.36 a	116.17 ± 3.76 a	113.50 ± 11.84 a	117.50 ± 5.01 a	116.67 ± 4.8 a	8
Mean Corpuscular Volume (MCV)	0 mg/kg	52.82 ± 2.21 b	50.68 ± 3.64 ab	54.64 ± 1.35 b	54.95 ± 2.04 a	52.82 ± 2.21 a	8
	20 mg/kg	51.35 ± 3.52 ab	50.89 ± 3.23 ab	50.15 ± 1.84 b	50.06 ± 3.89 b	54.35 ± 3.08 a	8
	25 mg/kg	52.11 ± 2.08 BC	50.97 ± 3.77 BC	50.55 ± 2.17 C	53.63 ± 2.32 AB	55.55 ± 2.45 A	8
	30 mg/kg	50.67 ± 2.62 a	51.79 ± 2.85 a	50.30 ± 4.55 a	52.96 ± 5.00 a	54.01 ± 3.14 a	8
Lymphocyte Count (Lym#)	0 mg/kg	10.16 ± 5.43 a	10.18 ± 3.94 a	12.14 ± 5.90 a	9.30 ± 4.54 a	9.28 ± 4.39 a	8
	20 mg/kg	10.19 ± 9.78 a	9.49 ± 6.23 a	6.56 ± 4.24 a	10.65 ± 4.41 a	9.95 ± 6.63 a	8
	25 mg/kg	10.40 ± 4.47 ab	11.13 ± 6.77 ab	5.33 ± 2.11 b	12.66 ± 5.03 a	9.47 ± 4.08 a	8
	30 mg/kg	10.16 ± 5.13 a	10.12 ± 6.34 a	5.38 ± 3.70 a	7.80 ± 8.18 a	7.01 ± 6.24 a	8
Mid-Cell Count (Mid#)	0 mg/kg	1.16 ± 0.13 a	0.95 ± 0.31 a	1.17 ± 0.25 a	1.17 ± 0.20 a	1.25 ± 0.23 a	8
	20 mg/kg	0.99 ± 0.27 a	1.00 ± 0.43 a	0.90 ± 0.09 a	1.11 ± 0.36 a	1.04 ± 0.34 a	8
	25 mg/kg	0.86 ± 0.18 B	0.84 ± 0.19 B	0.82 ± 0.23 B	1.10 ± 0.28 A	1.06 ± 0.24 AB	8
	30 mg/kg	1.08 ± 0.32 a	0.93 ± 0.57 a	1.00 ± 0.81 a	1.11 ± 0.80 a	1.29 ± 0.64 a	8
Lymphocyte Percentage (Lym%)	0 mg/kg	72.22 ± 4.56 a	77.42 ± 7.19 a	72.10 ± 8.97 a	75.70 ± 10.32 a	72.22 ± 4.56 a	8
	20 mg/kg	75.58 ± 9.82 a	75.91 ± 14.03 a	72.82 ± 8.93 a	75.08 ± 7.39 a	75.67 ± 11.31 a	8
	25 mg/kg	76.81 ± 11.31 a	78.80 ± 7.89 a	74.51 ± 4.55 a	77.65 ± 6.62 a	73.18 ± 3.51 a	8
	30 mg/kg	75.83 ± 14.44 a	74.31 ± 12.17 a	75.99 ± 15.01 a	75.78 ± 10.48 a	70.73 ± 12.78 a	8
Mid-Cell Percentage (Mid%)	0 mg/kg	8.83 ± 3.04 B	8.66 ± 1.71 B	7.00 ± 2.36 B	10.32 ± 3.33 B	14.10 ± 1.94 A	8
	20 mg/kg	8.56 ± 4.19 a	8.40 ± 3.49 a	9.25 ± 3.05 a	9.59 ± 3.17 a	8.74 ± 4.41 a	8
	25 mg/kg	7.86 ± 3.99 a	8.55 ± 5.42 a	8.73 ± 1.55 a	9.35 ± 3.43 a	9.86 ± 2.89 a	8
	30 mg/kg	8.53 ± 4.65 a	8.65 ± 4.42 a	8.38 ± 3.97 a	9.64 ± 4.21 a	11.93 ± 5.22 a	8

Different uppercase letters in the same row indicate significant differences (*p* < 0.05); different lowercase letters in the same row indicate a trend toward significance (0.05 ≤ *p* < 0.1); same lowercase letters in the same row indicate no significant difference (*p* > 0.05). In this study, the 0 mg/kg group served as the control, providing the baseline normal physiological reference values for this breed under standardized rearing conditions.

**Table 4:** Effects of cyclophosphamide on creatine kinase content, creatine kinase activity, and myoglobin in sheep.

Days Post- Administration	Dose	Creatine Kinase (CK) Content (ng/mL)	Creatine Kinase (CK) Activity (IU/L)	Myoglobin (MYO/MB) (ng/mL)	Number of Sheep (Group)
3D	25 mg/kg	19.28 ± 0.91 B	24.47 ± 1.89 B	175.48 ± 9.93 B	6
15D	25 mg/kg	43.58 ± 1.80 A	60.06 ± 1.21 A	348.21 ± 13.44 A	6
30D	25 mg/kg	17.01 ± 0.99 C	21.13 ± 1.02 C	168.04 ± 9.73 B	6
0D	0 mg/kg	18.56 ± 1.20 B	23.53 ± 1.80 B	179.40 ± 10.98 B	6

Different uppercase letters in the same column indicate significant differences (*p* < 0.05). In this study, the 0 mg/kg group served as the control, providing the baseline normal physiological reference values for this breed under standardized rearing conditions.

**Table 5:** Effects of cyclophosphamide on carcass characteristics in sheep.

Dose	Days Post-Administration	Pre-Slaughter Live Weight	Carcass Weight	Dressing Percentage	Number of Sheep (Group)
25 mg/kg	3D	23.67 ± 2.92	10.17 ± 0.82	0.43 ± 0.02	6
25 mg/kg	15D	24.90 ± 3.93	11.53 ± 2.46	0.47 ± 0.03	6
25 mg/kg	30D	22.30 ± 1.07	10.22 ± 0.79	0.46 ± 0.02	6
0 mg/kg	0D	23.73 ± 1.93	10.85 ± 1.08	0.46 ± 0.02	6

In this study, the 0 mg/kg group served as the control, providing the baseline normal physiological reference values for this breed under standardized rearing conditions.

**Table 6:** Effects of cyclophosphamide on meat quality in sheep.

Parameter	Experimental Group (25 mg/kg)	Experimental Group (25 mg/kg)	Experimental Group (25 mg/kg)	Control Group (0 mg/kg)	Number of Sheep (Group)
Days Post-Administration	3D	15D	30D	0D	/
Moisture Content (%)	0.74 ± 0.03 a	0.75 ± 0.02 a	0.73 ± 0.05 a	0.76 ± 0.01 a	6
Crude Protein (g/100 g fresh weight)	24.00 ± 1.90 a	24.53 ± 1.18 a	25.28 ± 1.20 a	24.31 ± 1.19 a	6
Crude Fat (g/100 g fresh weight)	10.20 ± 7.41 a	10.71 ± 3.95 a	9.18 ± 4.51 a	11.20 ± 3.65 a	6
pH value	5.27 ± 0.14 C	5.54 ± 0.25 B	5.82 ± 0.25 A	5.46 ± 0.16 BC	6
Shear Force (gf)	1241.76 ± 171.01 a	1082.28 ± 172.87 a	1300.00 ± 173.39 a	1249.12 ± 122.57 a	6
Marbling Score	2.50 ± 0.19 b	2.95 ± 0.39 a	2.67 ± 0.21 ab	2.50 ± 0.19 b	6
Water Holding Capacity (%)	96.48 ± 0.70 ab	96.98 ± 0.77 a	96.02 ± 0.16 b	96.54 ± 0.62 ab	6
Color Grade	3.11 ± 0.17 a	3.11 ± 0.34 a	3.17 ± 0.18 a	3.11 ± 0.17 a	6
Lightness (L*)	48.90 ± 0.37 a	48.90 ± 0.21 a	48.83 ± 0.19 a	48.86 ± 0.37 a	6
Redness (a*)	15.57 ± 0.37 a	15.80 ± 0.38 a	15.59 ± 0.48 a	15.99 ± 0.31 a	6
Yellowness (b*)	9.04 ± 0.35 B	9.51 ± 0.11 A	9.37 ± 0.21 A	9.01 ± 0.24 B	6

Different uppercase letters in the same row indicate significant differences (*p* < 0.05); different lowercase letters in the same row indicate a trend toward significance (0.05 ≤ *p* < 0.1); same lowercase letters in the same row indicate no significant difference (*p* > 0.05). In this study, the 0 mg/kg group served as the control, providing the baseline normal physiological reference values for this breed under standardized rearing conditions.

**Table 7:** Effects of cyclophosphamide on amino acid content in sheep.

Parameter (μg/g)	Experimental Group (25 mg/kg)	Experimental Group (25 mg/kg)	Experimental Group (25 mg/kg)	Control Group (0 mg/kg)	Number of Sheep (Group)
Days Post-Administration	3D	15D	30D	0D	/
Valine (Val)	21.64 ± 3.54 a	19.75 ± 2.37 a	19.05 ± 2.98 a	21.90 ± 3.86 a	6
Threonine (Thr)	15.79 ± 2.93 a	13.96 ± 1.56 a	13.68 ± 0.41 a	14.68 ± 2.52 a	6
Isoleucine (Ile)	17.92 ± 6.34 a	19.10 ± 2.21 a	16.55 ± 1.87 a	21.16 ± 2.92 a	6
Lysine (Lys)	20.09 ± 4.82 b	21.43 ± 3.67 ab	22.98 ± 5.42 ab	28.25 ± 7.51 a	6
Methionine (Met)	12.55 ± 7.07 a	13.15 ± 2.60 a	10.93 ± 4.13 a	15.72 ± 4.93 a	6
Phenylalanine (Phe)	27.79 ± 14.48 ab	27.09 ± 4.91 ab	19.08 ± 2.78 b	35.31 ± 9.65 a	6
Leucine (Leu)	48.03 ± 17.34 a	43.15 ± 7.10 a	38.27 ± 7.46 a	55.98 ± 16.28 a	6
Tryptophan (Trp)	5.33 ± 2.56 a	5.43 ± 1.12 a	4.78 ± 0.66 a	5.81 ± 0.98 a	6
EAA	169.14 ± 59.08 a	163.06 ± 25.54 a	145.32 ± 25.71 a	198.81 ± 48.65 a	6
Glycine (Gly)	247.37 ± 90.92 a	195.21 ± 63.35 a	168.80 ± 10.85 a	256.67 ± 84.90 a	6
Alanine (Ala)	141.92 ± 16.02 a	156.59 ± 28.75 a	157.72 ± 26.27 a	146.70 ± 42.18 a	6
γ-Aminobutyric Acid (GABA)	2.19 ± 1.33 a	2.10 ± 0.46 a	2.28 ± 1.17 a	2.29 ± 0.49 a	6
Serine (Ser)	48.52 ± 18.37 ab	34.25 ± 7.90 b	37.93 ± 6.98 b	66.16 ± 19.53 a	6
Proline (Pro)	14.50 ± 1.76 a	14.12 ± 2.23 a	13.45 ± 2.21 a	13.57 ± 3.68 a	6
Aspartic Acid (Asp)	25.93 ± 11.65 a	22.65 ± 5.53 a	26.43 ± 6.67 a	22.52 ± 16.26 a	6
Arginine (Arg)	23.86 ± 3.77 a	23.54 ± 4.93 a	20.99 ± 6.33 a	24.83 ± 5.04 a	6
Histidine (His)	281.55 ± 119.17 a	371.96 ± 71.28 a	249.45 ± 143.81 a	256.66 ± 104.14 a	6
Asparagine (Asn)	9.05 ± 4.03 a	8.40 ± 0.96 a	8.31 ± 2.15 a	9.51 ± 3.69 a	6
Tyrosine (Tyr)	23.75 ± 8.57 a	25.47 ± 4.33 a	20.50 ± 10.01 a	27.36 ± 6.80 a	6
Glutamic Acid (Glu)	35.32 ± 11.92 a	14.90 ± 4.29 c	19.32 ± 8.64 bc	41.79 ± 21.03 a	6
NEAA	853.96 ± 287.51 a	869.19 ± 194.01 a	725.18 ± 225.09 a	868.06 ± 307.74 a	/
EAA/NEAA	0.20 ± 0.21 a	0.19 ± 0.13 a	0.20 ± 0.11 a	0.23 ± 0.16 a	/

Different lowercase letters in the same row indicate a trend toward significance (0.05 ≤ *p* < 0.1); same lowercase letters in the same row indicate no significant difference (*p* > 0.05). In this study, the 0 mg/kg group served as the control, providing the baseline normal physiological reference values for this breed under standardized rearing conditions.

**Table 8:** Effects of cyclophosphamide on saturated fatty acid content in sheep.

Parameter (μg/g)	Experimental Group (25 mg/kg)	Experimental Group (25 mg/kg)	Experimental Group (25 mg/kg)	Control Group (0 mg/kg)	Number of Sheep (Group)
Days	3D	15D	30D	30D	
C6:0	0.40 ± 0.04 a	0.49 ± 0.14 a	0.41 ± 0.14 a	0.39 ± 0.28 a	6
C8:0	0.46 ± 0.19 a	0.56 ± 0.23 a	0.41 ± 0.16 a	0.43 ± 0.14 a	6
C10:0	3.54 ± 3.26 a	3.80 ± 2.40 a	2.44 ± 1.41 a	2.48 ± 1.83 a	6
C11:0	0.81 ± 0.04 a	0.83 ± 0.05 a	0.79 ± 0.03 a	0.78 ± 0.03 a	6
C12:0	2.46 ± 1.07 a	2.92 ± 1.25 a	2.61 ± 0.91 a	2.44 ± 1.71 a	6
C13:0	1.16 ± 0.11 a	1.11 ± 0.12 a	1.10 ± 0.09 a	1.16 ± 0.22 a	6
C14:0	38.97 ± 25.52 a	45.75 ± 25.83 a	36.30 ± 17.86 a	36.37 ± 27.21 a	6
C15:0	7.83 ± 3.32 a	7.32 ± 3.22 a	7.17 ± 3.16 a	7.86 ± 3.48 a	6
C16:0	495.34 ± 336.77 a	543.92 ± 204.99 a	491.09 ± 317.42 a	504.87 ± 363.72 a	6
C17:0	30.27 ± 11.20 a	28.80 ± 8.72 a	23.26 ± 8.31 a	24.48 ± 4.39 a	6
C18:0	545.92 ± 335.37 a	490.97 ± 221.08 a	373.37 ± 287.05 a	329.39 ± 82.86 a	6
C20:0	11.50 ± 1.31 a	10.82 ± 0.59 a	10.65 ± 0.80 a	10.81 ± 0.65 a	6
C21:0	9.36 ± 0.12 a	9.33 ± 0.16 a	9.19 ± 0.06 a	9.19 ± 0.12 a	6
C22:0	0.75 ± 0.19 a	0.66 ± 0.11 ab	0.60 ± 0.09 b	0.45 ± 0.07 a	6
C24:0	4.47 ± 0.18 b	4.31 ± 0.11 b	4.26 ± 0.05 b	4.2 ± 0.09 a	6
∑SFA	1153.24 ± 718.69 a	1151.59 ± 469.00 a	963.65 ± 637.54 a	935.30 ± 486.80 a	/

Different lowercase letters in the same row indicate a trend toward significance (0.05 ≤ *p* < 0.1); same lowercase letters in the same row indicate no significant difference (*p* > 0.05). In this study, the 0 mg/kg group served as the control, providing the baseline normal physiological reference values for this breed under standardized rearing conditions.

**Table 9:** Effects of cyclophosphamide on monounsaturated fatty acid content in sheep.

Parameter (μg/g)	Experimental Group (25 mg/kg)	Experimental Group (25 mg/kg)	Experimental Group (25 mg/kg)	Control Group (0 mg/kg)	Number of Sheep (Group)
Days	3D	15D	30D	30D	/
C14:1T	2.45 ± 1.02 ab	1.70 ± 0.60 ab	1.53 ± 0.53 b	1.09 ± 0.51 a	6
C14:1	2.36 ± 0.78 a	2.17 ± 0.63 a	1.49 ± 0.33 a	1.46 ± 0.81 a	6
C15:1T	3.05 ± 0.76 a	2.35 ± 0.84 a	2.41 ± 0.78 a	2.48 ± 1.24 a	6
C15:1	2.54 ± 0.48 B	1.85 ± 0.13 BC	1.53 ± 0.27 C	1.25 ± 0.13 A	6
C16:1T	6.38 ± 3.47 a	5.85 ± 3.18 a	5.39 ± 2.84 a	5.92 ± 4.25 a	6
C16:1	26.60 ± 17.81 a	34.37 ± 17.39 a	25.19 ± 16.26 a	22.14 ± 6.55 a	6
C17:1T	4.92 ± 2.83 a	3.99 ± 1.63 a	3.80 ± 2.06 a	2.64 ± 0.99 a	6
C17:1	14.20 ± 2.83 a	14.73 ± 3.26 a	13.61 ± 2.88 a	13.09 ± 2.25 a	6
C18:1N7T	14.80 ± 7.82 a	15.33 ± 10.14 a	9.76 ± 5.98 a	9.36 ± 3.16 a	6
C18:1N9C	608.18 ± 434.79 a	618.16 ± 152.41 a	418.70 ± 360.08 a	439.71 ± 142.77 a	6
C18:1N7	27.32 ± 12.32 a	31.43 ± 8.92 a	24.80 ± 12.39 a	20.55 ± 5.49 a	6
C19:1N9T	4.19 ± 0.99 a	4.05 ± 0.32 a	3.29 ± 0.40 a	3.41 ± 0.79 a	6
C20:1T	5.85 ± 0.50 B	5.34 ± 0.11 C	4.83 ± 0.26 D	4.40 ± 0.07 A	6
C20:1	13.00 ± 1.64 a	12.47 ± 0.74 b	11.43 ± 0.43 b	11.38 ± 0.52 a	6
C22:1N9T	10.66 ± 0.53 A	10.28 ± 0.23 B	9.62 ± 0.25 B	9.10 ± 0.48 A	6
C22:1N9	11.23 ± 0.43 A	10.95 ± 0.45 B	10.27 ± 0.15 C	9.71 ± 0.31 A	6
C24:1	2.49 ± 1.27 ab	1.19 ± 0.58 b	0.47 ± 0.35 b	0.51 ± 0.55 a	6
∑MUFA	760.22 ± 490.27 a	776.21 ± 201.56 a	548.12 ± 406.24 a	558.2 ± 170.87 a	/

Different uppercase letters in the same row indicate significant differences (*p* < 0.05); different lowercase letters in the same row indicate a trend toward significance (0.05 ≤ *p* < 0.1); same lowercase letters in the same row indicate no significant difference (*p* > 0.05). In this study, the 0 mg/kg group served as the control, providing the baseline normal physiological reference values for this breed under standardized rearing conditions.

**Table 10:** Effects of cyclophosphamide on polyunsaturated fatty acid content in sheep.

Parameter (μg/g)	Experimental Group (25 mg/kg)	Experimental Group (25 mg/kg)	Experimental Group (25 mg/kg)	Control Group (0 mg/kg)	Number of Sheep (Group)
Days	3D	15D	30D	30D	/
C18:2N6T	9.81 ± 1.44 a	9.69 ± 0.86 a	9.18 ± 1.07 a	9.15 ± 1.13 a	6
C18:2N6	113.78 ± 33.94 a	112.93 ± 27.98 a	101.83 ± 19.09 a	105.16 ± 36.41 a	6
C18:3N6	12.61 ± 0.78 a	12.44 ± 0.4 b	11.56 ± 0.31 b	11.62 ± 0.45 a	6
C18:3N3	20.38 ± 3.92 a	19.12 ± 2.14 a	18.45 ± 2.31 a	18.19 ± 2.45 a	6
C20:2	14.28 ± 0.82 A	13.98 ± 0.35 B	13.28 ± 0.2 B	13.03 ± 0.27 A	6
C20:3N6	22.61 ± 1.93 ab	21.86 ± 0.61 b	21.1 ± 0.34 b	20.6 ± 0.55 a	6
C20:3N3	19.99 ± 0.51 AB	19.66 ± 0.23 BC	19.28 ± 0.3 C	19.03 ± 0.21 A	6
C20:4N6	66.05 ± 17.29 a	63.87 ± 5.89 a	59.03 ± 11.12 a	51.38 ± 4 a	6
C22:2	50.61 ± 0.74 ab	50.04 ± 0.62 b	49.37 ± 0.41 b	49.39 ± 0.59 a	6
C20:5N3	23.16 ± 1.63 ab	22.19 ± 1.14 ab	21.84 ± 1.19 b	20.92 ± 0.42 a	6
C22:4	13.3 ± 2.02 a	12.58 ± 0.89 a	11.81 ± 0.77 a	12.07 ± 0.62 a	6
∑PUFA	366.58 ± 65.02 a	358.36 ± 41.11 a	336.73 ± 37.11 a	330.54 ± 47.1 a	/

Different uppercase letters in the same row indicate significant differences (*p* < 0.05); different lowercase letters in the same row indicate a trend toward significance (0.05 ≤ *p* < 0.1); same lowercase letters in the same row indicate no significant difference (*p* > 0.05). In this study, the 0 mg/kg group served as the control, providing the baseline normal physiological reference values for this breed under standardized rearing conditions.

## Data Availability

The raw data supporting the conclusions of this article will be made available by the authors on request.

## Abbreviations

The following abbreviations are used in this manuscript:

CPA	Cyclophosphamide
CK	Creatine kinase
HGB	Hemoglobin
Lym	Lymphocyte
MCV	Mean corpuscular volume
RBC	Red blood cell
WBC	White blood cell
